# Higher recreational screen time and lower step count are associated with higher cardiovascular disease risk in early adolescence

**DOI:** 10.1186/s12889-026-26756-z

**Published:** 2026-03-02

**Authors:** Jason M. Nagata, Nathan D. Nguyen, Isaac Frimpong, Alexander Heuer, Christiane K. Helmer, Abubakr A. Al-Shoaibi, Kyle T. Ganson, Alexander Testa, Erin E. Dooley, Thomas P. Le, Kelley Pettee Gabriel, Fiona C. Baker, Holly C. Gooding

**Affiliations:** 1 Division of Adolescent and Young Adult Medicine, Department of Pediatrics, University of California, San Francisco, Box 0503, 550 16th Street, San Francisco, CA 94143 USA; 2 Factor-Inwentash Faculty of Social Work, University of Toronto, 246 Bloor St W, Toronto, ON M5S 1V4 Canada; 3 Department of Management, Policy and Community Health, University of Texas Health Science Center at Houston, 7000 Fannin St, Houston, TX 77030 USA; 4 Department of Epidemiology, University of Alabama at Birmingham, 1665 University Boulevard, Birmingham, AL 35233 USA; 5 Department of Psychology, Bryn Mawr College, 151 N Merion Ave, Bryn Mawr, PA 19010 USA; 6 Center for Health Sciences, SRI International, Menlo Park, 333 Ravenswood Ave, Menlo Park, CA 94025 USA; 7 Division of General Pediatrics and Adolescent Medicine, Department of Pediatrics, Emory University School of Medicine, James B. Williams Medical Education Building, 100 Woodruff Circle, Atlanta, GA 30322 USA

**Keywords:** Youth, Teen, Exercise, Metabolic health, Vascular health, Blood lipids, Digital media, Prevention

## Abstract

**Background:**

Adolescence is a critical period for developing behaviors that affect lifelong cardiovascular health. While physical activity improves cardiovascular outcomes, excessive recreational screen time is linked to negative cardiovascular indicators. This study examined associations between screen time and physical activity with cardiovascular risk factors in a large, diverse sample of early adolescents.

**Methods:**

This study included 4,443 adolescents from the Adolescent Brain Cognitive Development (ABCD) Study collected at Year 2 (2018–2020) and Year 4 (2020–2022). Screen time was self-reported and categorized as low (0–4), medium (> 4–8), or high (> 8) hours/day. Physical activity was measured via Fitbit step count over 3 weeks and categorized as low (1,000–6,000), medium (> 6,000–12,000), and high (> 12,000) steps/day. Outcomes included blood pressure percentile and hypertensive-range blood pressure, which were available for the full sample, while total cholesterol, high-density lipoprotein (HDL) cholesterol, non-HDL cholesterol, hemoglobin A1c, and testing consistent with diabetes were available in a subset (one-third) at Year 4. Adjusted linear and Poisson regression models examined joint associations of screen time and step count with cardiovascular health outcomes.

**Results:**

Adolescents averaged 6.1 (± 5.1) hours/day of screen use and 9,280 (± 3,279) steps/day. Higher screen time and lower step count showed dose-response associations with higher diastolic blood pressure percentile (all *p* < 0.01). Compared to reference categories, high screen time (> 8 h/day) was associated with a 4.25-point increase in diastolic blood pressure percentile (95% confidence interval [CI] 1.85 to 6.65) and low step count (1,000–6,000 steps/day) was associated with a 7.15-point increase (95% CI 4.82 to 9.48). Both high screen time (adjusted risk ratio [ARR] 1.88, 95% CI 1.42 to 2.56) and low step count (ARR 2.35, 95% CI 1.88 to 2.94) were associated with increased risk of hypertensive-range blood pressure. Low step count was associated with -3.72 mg/dL lower HDL cholesterol (95% CI -6.52 to -2.92) and increased risk of low HDL cholesterol (ARR 2.05, 95% CI 1.79 to 2.36).

**Conclusions:**

Higher screen time and lower physical activity were associated with poorer cardiovascular health, supporting guidelines and early interventions promoting increased physical activity and reduced screen time.

**Supplementary Information:**

The online version contains supplementary material available at 10.1186/s12889-026-26756-z.

## Introduction

The American Heart Association identifies several key metrics of cardiovascular health, including blood cholesterol levels (high-density lipoprotein [HDL] cholesterol and low-density lipoprotein [LDL] cholesterol), body mass index (BMI), blood glucose, and blood pressure [1]. These factors collectively contribute to cardiovascular disease (CVD) risk, which is the leading contributor to premature death and disability in U.S. adults [2]. Because lifestyle behaviors such as physical activity and screen time established during adolescence often persist into adulthood, this developmental stage represents a critical window for CVD prevention [3].

Daily physical activity and higher levels of cardiorespiratory fitness are associated with better adolescent cardiovascular health outcomes [4], mental health [5], and academic achievement [6]. The 2018 *Physical Activity Guidelines for Americans*, issued by the U.S. Department of Health and Human Services, recommend that children and adolescents aged 6–17 years engage in at least 60 min of moderate-to-vigorous intensity physical activity daily [7]. However, previous studies reveal that most U.S. youth fall short of this target [8, 9]. The 2024 U.S. Report Card on Physical Activity for Children and Youth shows that less than 28% of children and adolescents meet the daily recommendation, representing a decline since 2016 [10].

Simultaneously, recreational screen time among adolescents has increased substantially [11–13]. In 2016, U.S. adolescents spent an average of 4–6 h per day on digital media [14, 15]. By 2024, the U.S. Surgeon General’s Advisory on Social Media and Youth Mental Health reported that one in four adolescents exceeded 5 h of daily screen time [16]. Excessive screen time has been linked to negative impacts on cardiovascular health indicators, including increased blood pressure [17], non-HDL cholesterol [18], adiposity, insulin resistance, and risk of diabetes [19, 20] in children and adolescents. Screen time is a major contributor to sedentary behavior during discretionary time as it often involves prolonged periods of physical inactivity across activities such as watching TV shows or movies, texting, using social media, or gaming. Previous research from the cross-sectional HELENA study found that more than two hours per day of sedentary behavior was associated with increased cardiometabolic risk [21]. Physiologically, sedentary behavior represents a state of low energy expenditure that can adversely affect several biological mechanisms, including metabolic dysfunction, insulin resistance, vascular injury, increased body fat, decreased cardiovascular fitness, and elevated inflammatory markers [22].

While previous research shows that decreased physical activity and increased screen time are associated with a higher risk of CVD [23], the *2018 Physical Activity Guidelines Advisory Committee Scientific Report* suggests the need for further research on the dose-response relationships of screen time and physical activity on cardiovascular health outcomes in adolescents [24]. Current evidence is limited regarding how to best guide physical activity and screen time recommendations for adolescents.

Few studies have examined the combined associations of screen time and physical activity on markers of cardiovascular health in adolescents. A prior cross-sectional analysis of the Adolescent Brain Cognitive Development (ABCD) Study found that adolescents with lower screen time and higher step counts had lower CVD risk, including lower diastolic blood pressure and higher HDL cholesterol [25]. The present study extends these findings by including a study design with two-year follow-up, allowing for better delineation of temporal relationships through multiple waves of data collection, and by further examining joint associations of physical activity (daily step count) and screen time exposures with CVD risk factors (blood pressure, hemoglobin A1c, and cholesterol levels).

We hypothesize that, during adolescence, lower recorded step count and higher self-reported screen time will be associated with higher risk of CVD risk factors two years later.

## Methods

We used data from Year 2 (2018–2020) and Year 4 (2020–2022) follow-ups of the longitudinal ABCD Study from the 6.0 data release. The ABCD Study was initiated between 2016 and 2018 (baseline) and is the largest long-term assessment of adolescent brain development and health in the U.S. The study recruited 11,875 children with diverse racial, ethnic, and socioeconomic backgrounds from 21 sites across the U.S. Previous investigations have detailed the study’s design, recruitment methods, data collection, and procedures [26]. Among participants with available data, complete exposure data (both screen time and Fitbit physical activity measures) were available for 9,023 participants, with 2,939 participants excluded for missing either screen time or Fitbit data. Among those with complete exposure data, 6,511 participants had complete sociodemographic covariate data, with 2,512 participants excluded for missing baseline demographic variables (household income, parental education, race/ethnicity, or parent marital status). An additional 12 participants were excluded for missing BMI data. The final analytical sample of 4,443 participants had complete exposure, covariate, and blood pressure outcome data, with 2,056 participants excluded for missing blood pressure measurements. Blood samples were collected from a subset of participants who consented to blood draws at Year 4. For analyses involving blood-based cardiovascular markers, sample sizes varied based on data availability: hemoglobin A1c (*n* = 1,571), total cholesterol (*n* = 1,627), and HDL cholesterol (*n* = 1,651), reflecting participants who provided blood samples at Year 4 follow-up. A flow diagram summarizing participant inclusion and exclusion is shown in Supplemental Figure S1, and differences between included and excluded participants are presented in Supplemental Table S1. Institutional review board (IRB) approval was obtained at the University of California, San Diego (UCSD) and each study site. Participants provided written assent and caregivers provided written informed consent.

### Exposure variables (Year 2)

All exposure data was extracted from the ABCD Study Year 2 (November 2018 to November 2020).

#### Screen time

Self-reported screen time data was collected via the Screen Time Questionnaire, which asked adolescents to estimate the total number of hours that they spent engaging with screens recreationally (e.g. watching television and videos, playing video games, texting, social media, and video chat) per day on weekdays and weekends, separately. The total screen use weighted average was calculated with the formula: ([weekday average x 5] + [weekend average x 2])/7. Screen time was categorized as low (0–4 h/day), medium (4–8 h/day), or high (greater than 8 h/day). There are currently no standard screen time cutoffs, with the current American Academy of Pediatrics (AAP) removing the universal cutoff limits for adolescents [27, 28]. Therefore, our categorization was exploratory, based on prior studies identifying 4 h per day as a threshold linked to cardiovascular risk factors and metabolic dysfunction in adolescents [29, 30], and aligned with other national surveys using similar cutoffs [13]. Sensitivity analyses using screen time categories of 0–2, 2–4, and 4 + hours/day were also run.

#### Daily steps (Fitbit)

Participants enrolled in the ABCD Study were issued a Fitbit Charge series device (Fitbit Inc., San Francisco, CA) for a period of 21 days, during which their daily steps were measured. Data were available from 7,050 participants who enrolled in the optional Fitbit-based physical activity assessment during Year 2. Studies have shown good validity and feasibility of utilizing Fitbit devices to evaluate physical activity and daily step counts over time in adolescent populations. A study found that Fitbit assessment of physical activity, heart rate, and sleep were comparable to gold standard devices in adolescent populations [26]. Data were retrieved and interpreted using the ABCD Study guidelines [31, 32]. Based on recommendations from previous studies, only days with greater than 599 min of waking wear time and greater than 1,000 steps were included in the analysis. Data that met these criteria were further processed by grouping daily steps into low (1,000–6,000 steps), medium (6,000–12,000 steps), and high (greater than 12,000 steps) categories. These categorizations were based on the physical activity standards for adolescents, which recommend 60 min of moderate-to-vigorous intensity physical activity per day [7]. 12,000 steps meet these recommendations [29], and 6,000 steps represent half-dose of physical activity [34].

### Outcome variables (Year 4)

All outcome data were extracted from the ABCD Study Year 4 (November 2020 to November 2022), with binary outcomes representing the prevalence of each outcome at the Year 4 assessment.

#### Blood pressure percentile

ABCD Study research personnel received training on standardized protocols implemented across all sites. Prior to measurement, participants were seated in chairs for 5 min in quiet environments. Participants’ right arms were positioned palm-up on tables, with feet placed flat on the floor and legs uncrossed. Cuff sizing was determined through mid-upper arm circumference measurement. Three blood pressure measurements were obtained in 60-second intervals by researchers at each study site with a calibrated Omron blood pressure monitor (MicroLife USA, Inc; Dunedin, FL). Systolic and diastolic blood pressures were converted to age-, sex-, and height-based percentiles using AAP reference standards [35]. The prevalence of hypertensive-range blood pressure (secondary outcome) was defined as the proportion of participants with values at or above the 90th percentile according to pediatric guidelines for elevated blood pressure [35]. Participants receiving antihypertensive medications (*n* = 7) were excluded from blood pressure outcome analyses.

#### Hemoglobin A1c and diabetes

Hemoglobin A1c levels were assessed via venous blood collection as an indicator of average blood glucose levels over the preceding three months [33]. Participants with hemoglobin A1c levels ≥ 6.5% were classified as testing consistent with diabetes [36], and the prevalence was calculated as the proportion of participants meeting this criterion. Participants with a parent-reported history of diabetes (*n* = 11) were excluded from all hemoglobin A1c analyses to focus on incident diabetes risk and exclude those with presumed type 1 diabetes mellitus.

#### Total, HDL, and non-HDL cholesterol

Non-fasting total cholesterol and HDL cholesterol were assessed from the blood sample collected. Participants with a total cholesterol of greater than 200 mg/dL were categorized into the high total cholesterol (hyperlipidemia) outcome group, and participants with an HDL cholesterol of less than 40 mg/dL were categorized into the low HDL cholesterol outcome group [37]. Prevalence of high total cholesterol and low HDL cholesterol were assessed as secondary outcomes due to the low proportion of adolescents classified in these categories. Non–HDL cholesterol was calculated as total cholesterol minus HDL cholesterol, consistent with the recommendations of the Expert Panel on Integrated Guidelines for Cardiovascular Health and Risk Reduction in Children and Adolescents [38]. This measure captures all atherogenic lipoproteins, including low-density lipoprotein (LDL), very-low-density lipoprotein (VLDL), intermediate-density lipoprotein (IDL), and lipoprotein(a). The Expert Panel noted that it provides a more comprehensive assessment of cardiovascular risk than total cholesterol alone and remains a reliable indicator of atherogenic particle burden when fasting status is uncertain or when triglycerides are unavailable [38]. Furthermore, non-HDL cholesterol is recognized as a superior metric for assessing atherogenic lipid burden in clinical care [39]. The prevalence of high non-HDL cholesterol was defined as the percentage of participants with non-HDL cholesterol ≥ 145 mg/dL [40].

### Covariates

We used a causal inference framework to guide covariate selection, informed by a priori directed acyclic graphs (DAGs) [41, 42] based on existing literature; previous studies have shown that sex, racial and ethnic background, socioeconomic status, parent education level, and parent marital status are associated with screen time, physical activity, and cardiovascular health outcomes (blood pressure percentiles, hemoglobin A1c, total cholesterol, and HDL cholesterol) [14, 38, 43, 44]. Covariates included in the present analysis were Year 2 participant age, sex (female or male), race and ethnicity (Asian, Black, Latino/Hispanic, Native American, White, or other), household income (two categories reflecting the U.S. median household income: less than $75,000 and $75,000 or more [45]), parental education status (high school education or less vs. college education or more), parent marital status, and calendar month of data collection (to account for seasonal variation in physical activity). COVID-19 timing was also included as a covariate, with participants categorized as having Fitbit data collection before March 13, 2020 (pre-pandemic) versus during or after March 13, 2020 (pandemic period), as data collection may have been influenced by the COVID-19 pandemic and calendar month of data collection. Sensitivity analyses adjusting for BMI percentile (Supplemental Table S3) and waist circumference percentile (Supplemental Table S4) were conducted to assess robustness.

### Statistical analysis

Stata 18 (StataCorp, College Station, TX) was utilized for data analysis. First, we calculated descriptive statistics on extracted data from the study sample. Multivariable linear regression was used to examine associations between Year 2 screen time and step count with Year 4 CVD risk factors (systolic blood pressure percentile, diastolic blood pressure percentile, hemoglobin A1c, total cholesterol, and HDL cholesterol). Residual normality was confirmed using Shapiro-Wilk tests and Q-Q plots for all linear regression models. Modified Poisson regression with robust variance estimation was used to determine screen time and step count associations with binary cardiovascular prevalence outcomes (hypertensive-range blood pressure, diabetes, high total cholesterol, and low HDL cholesterol) [46]. This approach provides risk ratios (RRs), which are more appropriate for cohort data and avoid overestimation of associations [46].

Models used categorical predictors (screen time: low/medium/high; steps: low/medium/high). Statistical models were adjusted for age, sex, race and ethnicity, household income, parent education levels, parent marital status, and time of data collection (time of month and COVID-19 timing). Sensitivity analyses adjusted for the respective outcomes at Year 2 (blood pressure, hemoglobin A1c, and cholesterol data) in the subset of participants with available baseline measures (*n* = 238–272 depending on outcome) and are shown in Supplemental Table S2. Additional sensitivity analyses were conducted to assess the robustness of findings. These included analyses with adjustment for BMI percentile (Supplemental Table S3), analyses with adjustment for waist circumference percentile instead of BMI percentile as an alternative indicator of adiposity (Supplemental Table S4), analyses without adjustment for covariates that included only screen time and step count as joint independent variables (Supplemental Table S5), and analyses with alternate screen time categories (Supplemental Table S6).

For outcomes showing significant associations with both screen time and step categories, we created comprehensive nine-category exposure variables by cross-classifying all screen time categories (0–4, 4–8, > 8 h/day) with all step count categories (1k-6k, 6k-12k, > 12k steps/day) to examine joint associations, following a similar approach used in previous ABCD Study cardiovascular analyses [30]. We evaluated effect modification (interactions) between screen time and step categories for associations with each CVD risk factor outcome. Significance was indicated by a two-sided *p* < 0.05.

## Results

A total of 4,443 adolescents were included in this analysis. Overall, 48.5% of the participants were female and 38.8% were racial/ethnic minorities, with a mean age of 11.9 years at Year 2. Adolescents reported an average of 6.1 h of screen time per day. The average daily step count calculated across the Fitbit wear period was 9,280 steps per day. Mean systolic blood pressure percentile was 50.0 and mean diastolic blood pressure percentile was 46.0. Mean hemoglobin A1c was 5.2%, total cholesterol was 158.4 mg/dL, HDL cholesterol was 54.6 mg/dL, and non-HDL cholesterol was 103.8 mg/dL (Table 1).

**Table 1:** Sample characteristics of Adolescent Brain Cognitive Development (ABCD) Study participants at Year 2 included in the current analyses (N = 4,443)

Sociodemographic characteristics	Mean (95% CI)/n (%)
Age (years)	11.9 (11.93, 11.98)
Sex	
Female	2,157 (48.5%)
Male	2,286 (51.5%)
Race and ethnicity	
Asian	248 (5.6%)
Black	646 (14.5%)
Latino/Hispanic	631 (14.2%)
Native American	166 (3.7%)
White	2,721 (61.2%)
Other	31 (0.7%)
Household income	
Less than $75,000	2,915 (65.6%)
$75,000 or more	1,528 (34.4%)
Parent education	
High school education or less	389 (8.8%)
Some college education or more	4,054 (91.2%)
Parent marital status	
Parent married/partnered	3,489 (78.5%)
Parent not married/unpartnered	954 (21.5%)
Physical activity variables (step count per day)	
Total steps per day	9,280 (9,184, 9,376)
Step categories (%)	
1,000 to 6,000 steps per day	802 (18.1%)
6,000 to 12,000 steps per day	3,026 (68.1%)
>12,000 steps per day	615 (13.8%)
Screen time (hours per day)	
Total recreational screen time	6.1 (6.0, 6.2)
Screen time categories (%)	
0 to 4 hours per day	1,949 (43.9%)
4 to 8 hours per day	1,365 (30.7%)
>8 hours per day	1,129 (25.4%)
Cardiovascular disease risk measures (Year 4)	
Systolic blood pressure percentile (n=4,443)	50.0 (49.2, 50.8)
Diastolic blood pressure percentile (n=4,443)	46.0 (45.2, 46.8)
Hemoglobin A1c (%) (n = 1,571)	5.2 (5.18, 5.22)
Total cholesterol (mg/dL) (n = 1,627)	158.4 (156.9, 159.9)
HDL cholesterol (mg/dL) (n = 1,651)	54.6 (54.0, 55.2)
Non-HDL cholesterol (mg/dL) (n = 1,627)	103.8 (102.4, 105.2)

*CI* Confidence interval, *HDL* High-density lipoprotein

### Blood pressure percentile and hypertensive-range blood pressure

Higher screen time categories were associated with increased diastolic blood pressure percentile and risk of hypertensive-range blood pressure. Lower step count categories demonstrated dose-response associations with increased diastolic blood pressure percentile and hypertensive-range blood pressure risk, with low step count showing the strongest associations (Tables 2 and 3).

**Table 2:** Associations between self-reported total recreational screen time and step count categories and continuous cardiovascular disease (CVD) risk outcomes in the Adolescent Brain Cognitive Development (ABCD) Study

Screen time (hrs/day)	B (95% CI)	p	Steps/day	B (95% CI)	*p*
Systolic blood pressure percentile					
Low (0–4)	Reference		High (>12,000)	Reference	
Medium (4–8)	−0.25 (−2.42 to 1.92)	0.820	Medium (6,000–12,000)	**2.15 (0.93 to 2.82)**	**0.003**
High (>8)	0.95 (−1.52 to 3.42)	0.452	Low (1,000–6,000)	**3.52 (3.45 to 4.59)**	**0.018**
Diastolic blood pressure percentile					
Low (0–4)	Reference		High (>12,000)	Reference	
Medium (4–8)	**2.65 (−0.72 to 4.58)**	**0.007**	Medium (6,000–12,000)	**2.85 (0.95 to 4.75)**	**0.003**
High (>8)	**4.25 (1.85 to 6.65)**	**0.001**	Low (1,000–6,000)	**7.15 (4.82 to 9.48)**	**<0.001**
Hemoglobin A1c (%)					
Low (0–4)	Reference		High (>12,000)	Reference	
Medium (4–8)	−0.02 (−0.06 to 0.03)	0.528	Medium (6,000–12,000)	0.02 (−0.04 to 0.08)	0.475
High (>8)	−0.03 (−0.08 to 0.03)	0.385	Low (1,000–6,000)	0.05 (−0.02 to 0.12)	0.128
Total cholesterol (mg/dL)					
Low (0–4)	Reference		High (>12,000)	Reference	
Medium (4–8)	−3.42 (−8.15 to 1.31)	0.155	Medium (6,000–12,000)	−0.82 (−1.15 to 2.51)	0.760
High (>8)	−3.15 (−8.52 to 2.22)	0.248	Low (1,000–6,000)	−0.92 (−1.0 to 2.95)	0.782
HDL cholesterol (mg/dL)					
Low (0–4)	Reference		High (>12,000)	Reference	
Medium (4–8)	−0.30 (−2.25 to 1.65)	0.762	Medium (6,000–12,000)	−1.85 (−3.95 to 0.25)	0.084
High (>8)	−1.85 (−4.25 to 0.55)	0.131	Low (1,000–6,000)	**−3.72 (−6.52 to −2.92)**	**0.018**
Non-HDL cholesterol (mg/dL)					
Low (0–4)	Reference		High (>12,000)	Reference	
Medium (4–8)	1.05 (−0.15 to 4.45)	0.688	Medium (6,000–12,000)	−0.08 (−0.15 to 2.59)	0.648
High (>8)	**2.82 (1.85 to 6.49)**	**0.003**	Low (1,000–6,000)	**5.52 (3.35 to 8.69)**	**0.001**

All models include screen time and physical activity (step count) as the joint independent variables and were adjusted for Year 2 age, sex, race/ethnicity, household income, parental educational level, parental marital status, calendar month, data collection period (i.e., before or during the COVID-19 pandemic). Participants with a prior diagnosis of diabetes were excluded from the analysis of hemoglobin A1c and participants on hypertension medications were excluded from analyses of systolic and diastolic blood pressure

Bold indicates statistical significance at p<0.05

**Table 3:** Associations between self-reported total recreational screen time and step count categories and binary cardiovascular disease (CVD) risk outcomes in the Adolescent Brain Cognitive Development (ABCD) Study

Screen time (hrs/day)	ARR (95% CI	p	Steps/day	ARR (95% CI)	*p*
Hypertensive range - blood pressure (≥90th)					
Low (0–4)	Reference		High (>12,000)	Reference	
Medium (4–8)	**1.35 (1.08–1.95)**	**0.015**	Medium (6,000–12,000)	**1.75 (1.38–2.22)**	**0.001**
High (>8)	**1.88 (1.42–2.56)**	**<0.001**	Low (1,000–6,000)	**2.35 (1.88–2.94)**	**<0.001**
Testing consistent with diabetes (HbA1c ≥6.5%)					
Low (0–4)	Reference		High (>12,000)	Reference	
Medium (4–8)	0.57 (0.22–1.45)	0.235	Medium (6,000–12,000)	1.18 (0.84–5.10)	0.325
High (>8)	1.22 (0.93–3.15)	0.538	Low (1,000–6,000)	1.35 (0.78–2.35)	0.130
High total cholesterol (≥200 mg/dL)					
Low (0–4)	Reference		High (>12,000)	Reference	
Medium (4–8)	0.96 (0.74–1.88)	0.685	Medium (6,000–12,000)	1.02 (0.66–1.95)	0.445
High (>8)	1.58 (0.46–2.55)	0.455	Low (1,000–6,000)	1.75 (0.86–2.05)	0.725
Low HDL cholesterol (<40 mg/dL)					
Low (0–4)	Reference		High (>12,000)	Reference	
Medium (4–8)	1.25 (0.95–1.49)	0.078	Medium (6,000–12,000)	**1.45 (1.25–1.68)**	**<0.001**
High (>8)	**1.75 (1.50–2.04)**	**<0.001**	Low (1,000–6,000)	**2.05 (1.79–2.36)**	**<0.001**
High non-HDL cholesterol (≥145 mg/dL)					
Low (0–4)	Reference		High (>12,000)	Reference	
Medium (4–8)	1.18 (0.92–1.51)	0.195	Medium (6,000–12,000)	**1.28 (1.05–1.56)**	**0.015**
High (>8)	**1.55 (1.25–1.92)**	**0.045**	Low (1,000–6,000)	**1.85 (1.55–2.21)**	**<0.001**

All models include screen time and physical activity (step count) as the joint independent variables and were adjusted for age, sex, race/ethnicity, household income, parental educational level, parental marital status, calendar month, data collection period (i.e., before or during the COVID-19 pandemic). Participants with a prior diagnosis of diabetes were excluded from the analysis of hemoglobin A1c and participants on hypertension medications were excluded from analyses of systolic and diastolic blood pressure

Bold indicates statistical significance at p<0.05

### Hemoglobin A1c and diabetes

There were no significant associations between screen time and step count with hemoglobin A1c or testing consistent with diabetes (Tables 2 and 3).

### Total, HDL, and non-HDL cholesterol

High screen time and low step count were associated with elevated non-HDL cholesterol and increased risk of low HDL cholesterol (< 40 mg/dL) and high non-HDL cholesterol (≥ 145 mg/dL) (Tables 2 and 3). Low step count was also associated with lower HDL cholesterol (Table 2). There were no significant associations with total cholesterol.

### Interaction

There was no evidence of significant interactions between screen time and step categories for each of the CVD risk outcomes (all p for interaction > 0.05).

**Fig. 1:**
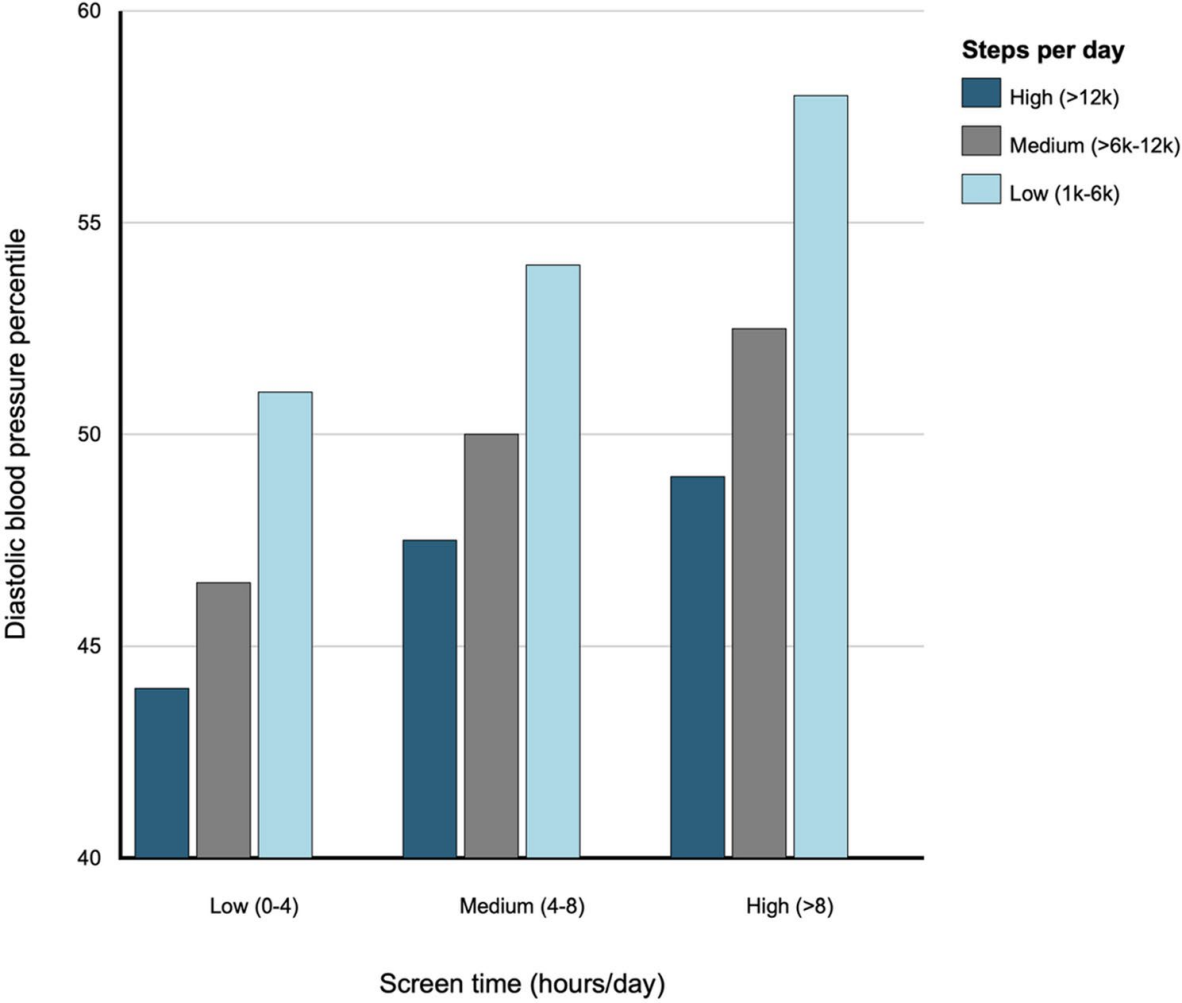
Associations between self-reported screen time and step count category combinations at Year 2 and diastolic blood pressure percentile at Year 4 in 4,443 participants of the Adolescent Brain Cognitive Development (ABCD) Study. Legend: Results correspond to coefficients from a multivariable linear regression model with nine categories of screen time and step combinations as the independent variable and diastolic blood pressure percentile as the dependent variable, adjusting for age, sex, race/ethnicity, household income, parental education, parent marital status, calendar month, and data collection period (i.e., before or during the COVID-19 pandemic) at Year 2. Daily step categories included: low (1,000–6,000), medium (6,000–12,000), and high (>12,000). Daily screen time categories (hours) included: low (0–4), medium (4–8), high (>8). The low screen time and high step categories were the reference categories

**Fig. 2:**
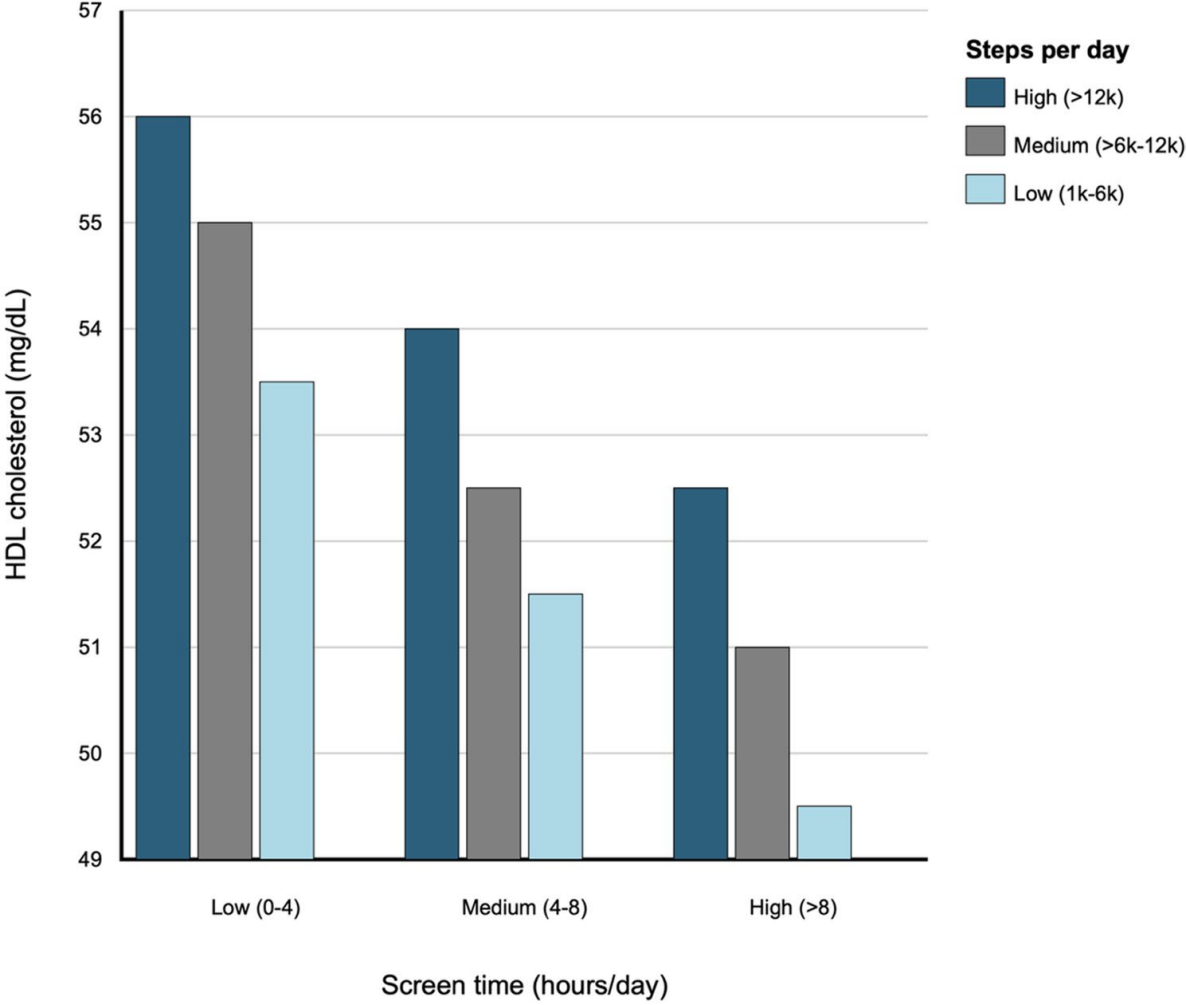
Associations between self-reported screen time and step count category combinations at Year 2 and HDL cholesterol at Year 4 in 4,443 participants of the Adolescent Brain Cognitive Development (ABCD) Study. Legend: Results correspond to coefficients from a linear regression model with nine categories of screen time and step combinations as the independent variable and HDL cholesterol as the dependent variable, adjusting for age, sex, race/ethnicity, household income, parental education, parent marital status, calendar month, and data collection period (i.e., before or during the COVID-19 pandemic) at Year 2. Daily step categories included: high (>12,000), medium (6,000–12,000), and low (1,000–6,000). Daily screen time categories (hours) included: low (0–4); medium (4–8), high (>8). The low screen time and high step categories was the reference categories

We further examined combinations of the 3 screen time and 3 step count categories (9 categories total) for diastolic blood pressure percentile and HDL cholesterol (Figs. 1 and 2). For diastolic blood pressure percentile, within each screen time category, lower step count categories were associated with higher diastolic blood pressure percentiles, ranging from approximately 3 to 14 percentile points higher compared to the reference category. For HDL cholesterol, within each screen time category, lower step count categories were associated with lower HDL cholesterol levels, ranging from approximately 1 to 6.5 mg/dL lower compared to the reference category. Results remained consistent in sensitivity analyses (Supplemental Figures S2 and S3).

### Sensitivity analyses

Compared to primary analyses, findings remained largely similar in models adjusted for Year 2 CVD risk factors (Supplemental Table S2), models adjusted for BMI percentile (Supplemental Table S3), models adjusted for waist circumference percentile (Supplemental Table S4), unadjusted models (Supplemental Table S5), and adjusted models using earlier (pre-2016) AAP screen time cutoffs (Supplemental Table S6).

## Discussion

In this study of a nationally diverse sample of U.S. adolescents from the ABCD Study, we examined the joint associations of recreational screen time and daily step count with the prevalence of key CVD risk factors two years later. Our findings revealed that high recreational screen time was associated with higher diastolic blood pressure, increased risk of hypertensive-range blood pressure, and higher non-HDL cholesterol, while low step count was associated with elevated diastolic blood pressure, increased risk of hypertensive-range blood pressure, lower HDL cholesterol, and higher non-HDL cholesterol two years later. Specifically, low step count was associated with the highest diastolic blood pressure percentile increase, followed by medium step count, indicating a clear dose-response relationship. We did not observe evidence of interaction between screen time and step count, suggesting independent and additive associations.

The lack of significant interactions indicates that high physical activity may not attenuate the association between excessive screen time and cardiovascular risk factors, consistent with established research showing these behaviors have independent associations [47, 48]. For example, a recent study found that sedentary behavior independent of physical activity behavior can induce adverse cardiometabolic effects such as insulin resistance, endothelial dysfunction, and impaired lipid metabolism, even when meeting physical activity recommendations [22]. This challenges the common assumption that exercise can compensate for sedentary time and suggests interventions should target both behaviors simultaneously rather than focusing on only one.

The current analysis builds on and confirms cross-sectional findings from our previous study, which reported associations between higher screen time and lower step count with higher diastolic blood pressure and lower HDL cholesterol [25]. These findings underscore the associations between adolescent behaviors and cardiovascular risk factors, highlighting the importance of developing early interventions for this population.

High screen time was associated with higher diastolic blood pressure and increased risk of hypertensive-range blood pressure, even when accounting for device-determined step count. In addition, having a lower daily step count was associated with higher diastolic blood pressure and increased risk of hypertensive-range blood pressure. This is consistent with findings in a previous cross-sectional analysis [25]. This is particularly important because previous studies have shown that diastolic blood pressure during adolescence is a contributor to adult CVD risk [49–51]. Recreational screen time is predominantly sedentary and may contribute to adverse cardiovascular outcomes by displacing physical activity and increasing caloric intake, particularly through passive behaviors such as snacking or exposure to unhealthy food advertisements online [52, 53]. Furthermore, higher levels of recreational screen time in adolescents have been associated with increased BMI percentile [25], a well-established risk factor for high blood pressure [54]. Physiologically, these associations may be explained by heightened sympathetic nervous system activity and early reductions in vascular function associated with sedentary behavior. While most mechanistic data are derived from adult studies, similar pathways are thought to occur in adolescents, which may be responsible for the observed elevations in diastolic blood pressure [22]. The physiological and behavioral mechanisms help explain the association between high screen time and adolescent cardiovascular health.

Screen time was not significantly associated with hemoglobin A1c or testing consistent with diabetes. These results are inconsistent with prior research demonstrating that sedentary behaviors, including prolonged screen time, are associated with insulin resistance, metabolic syndrome, and an elevated risk of type 2 diabetes in youth and adulthood [20, 55]. However, these studies are either cross-sectional or span adolescence into adulthood, whereas the present study examined changes in diabetes risk over a shorter two-year period during adolescence. Future research with longer follow-up durations and repeated measures may help clarify the association between screen time and diabetes risk across developmental stages.

We also found that high screen time and low daily step count were associated with higher non-HDL cholesterol, and that low daily step count was associated with lower HDL cholesterol, even after adjustment for recreational screen time. Specifically, we observed a dose-response relationship, with lower step count categories associated with lower HDL cholesterol.

These findings highlight the importance of physical activity as a behavioral factor in adolescent cardiovascular health. HDL cholesterol plays a key protective role in vascular health by facilitating reverse cholesterol transportation and reducing the accumulation of atherosclerotic plaque [56]. There is substantial evidence that dyslipidemia, including low HDL cholesterol levels, is associated with cardiovascular and metabolic disease in children and adolescents [56]. In our sample, the average daily step count was 9,280 steps, which is comparable with national estimates [57]. However, this average remains below the threshold of 12,000 steps per day, a suggested approximation of the recommended 60 min of moderate to vigorous intensity physical activity for adolescents [33]. These findings align with the results of the HELENA Study, which found that the combination of sedentary behavior less than two hours a day and physical activity levels of 60 min or greater is associated with low cardiometabolic risk among adolescents [21]. As cardiovascular risk factors often track from adolescence into adulthood, these results emphasize the need for early interventions that promote regular physical activity to support lifelong cardiovascular health [58].

Our study adds to the literature by indicating that both high recreational screen time and low physical activity are associated with increased adolescent CVD risk factors, specifically high diastolic blood pressure and low HDL cholesterol, two years later. We build on our previous cross-sectional investigation [25] by identifying these associations in a study design with two-year follow-up, strengthening the evidence for screen time and physical activity as independent behavioral targets for early CVD prevention.

These results also have implications in regard to the emerging clinical framework posited by the 2025 *Lancet Diabetes & Endocrinology Commission*, defining obesity as a chronic, systemic disease characterized by metabolic and organ dysfunction [59]. Within this framework, elevated diastolic blood pressure and reduced HDL cholesterol in adolescence with high screen time and low physical activity may indicate early metabolic dysfunction independent of overt obesity. Addressing both screen time and physical activity is important because both are associated with early pathophysiological markers related to cardiovascular and metabolic disease.

### Strengths and limitations

Strengths of this study include the study design with a two-year follow-up period, which allows for the assessment of temporal associations between screen time, step count, and CVD risk factors. Additionally, the large, racially and ethnically diverse population-based sample enhances the generalizability of our findings across U.S. adolescents. Objective measurement of physical activity using wearable Fitbit devices also provided a more precise and reliable estimate of daily movement compared to self-reported measures. The consistency of findings across different adiposity measures (BMI and waist circumference percentile) strengthens confidence in the robustness of the observed associations between screen time, physical activity, and cardiovascular health outcomes.

This study also has limitations. Although daily step count was objectively measured using Fitbit devices, data were collected over a 21-day period, which may not be representative of habitual or seasonal patterns over the course of a year. Although our analyses focused on daily step counts, steps capture only one aspect of physical activity. Their simplicity, however, makes them an intuitive and accessible target for prevention and behavior-change efforts. Future work should examine how step counts, alongside other activity dimensions, relate to cardiovascular health across development. Screen time was self-reported, potentially introducing recall errors and reporting bias, and did not assess for content or engagement intensity. As there are no standardized screen time cutoffs for adolescents, our categorization was based on prior literature and may limit comparability with studies using different thresholds.

Cardiometabolic blood measures such as blood pressure, cholesterol levels, and hemoglobin A1c were collected during a single visit two years after the initial assessment of screen time and physical activity and were only available for a subset of participants. Given the relatively short two-year follow-up period, we did not conduct formal incidence analyses, as this duration limits the ability to detect incident chronic cardiometabolic conditions and repeated cardiometabolic measurements were not available for all participants. Consequently, our findings reflect prevalent cardiometabolic risk at follow-up rather than disease incidence or progression, constraining causal inference and limiting conclusions regarding long-term risk trajectories. The follow-up interval may also be insufficient to capture the full extent of behavioral impacts on long-term cardiovascular outcomes, potentially leading to underestimated associations. Additionally, clinical measures obtained at a single time point may not accurately reflect participants’ typical cardiometabolic health, and the limited availability of blood-based measures restricts the generalizability of findings related to hemoglobin A1c and cholesterol. Furthermore, the ABCD Study does not collect data on triglycerides, which limits our ability to calculate LDL cholesterol and examine the relationships between screen time, physical activity, and both triglycerides and LDL cholesterol. Additionally, we did not examine whether race and ethnicity modified the associations between screen time, steps, and the CVD risk outcomes because the study was underpowered; larger samples will be needed to address this question. Finally, another limitation is the potential for unexamined confounders, although we controlled for sociodemographic factors and the COVID-19 pandemic.

## Conclusion

This cohort study contributes to the literature by identifying recreational screen time and physical activity thresholds that were associated with increased CVD risk in early adolescents. Among a diverse, population-based sample of U.S. adolescents, more than 8 h of recreational screen time per day and fewer than 6,000 steps per day were associated with higher diastolic blood pressure percentiles two years later. In addition, fewer than 12,000 steps per day were associated with lower HDL cholesterol levels. These findings underscore the need for early behavioral interventions and CVD prevention strategies that target adolescent screen time and physical activity. Currently, the U.S. Department of Health and Human Services’ 2018 *Physical Activity Guidelines for Americans* recommend at least 60 min of moderate to vigorous daily activity but provide no guidance on sedentary behavior, such as screen time [7]. Schools and communities should promote regular opportunities for physical activity, such as activity breaks during the school day, after-school sports or recreational programs, and safe environments for outdoor activities. Clinicians should also provide educational guidance on screen use to encourage families to set screen time limits and promote physically active alternatives. Future research should include more refined exposure measures, test for interaction by race and ethnicity, adopt longer-term longitudinal designs, assess incidence of CVD outcomes, and examine behavioral differences habitually and seasonally throughout the year.

## Supplementary Information

Supplementary Material 1

## Data Availability

Data used in the preparation of this article were obtained from the ABCD Study (https://abcdstudy.org), held in the NIH Brain Development Cohorts (NBDC) Portal.

## Abbreviations

ABCD: Adolescent Brain Cognitive Development Study
BMI: Body mass index
CVD: Cardiovascular disease
HDL: High-density lipoprotein cholesterol
LDL: Low-density lipoprotein cholesterol
