# Supplementary material for: Higher recreational screen time and lower step count are associated with higher cardiovascular disease risk in early adolescence
Source: BMC Public Health. 2026 Mar 2;26:1133. doi: 10.1186/s12889-026-26756-z (PMC13059598; doi:10.1186/s12889-026-26756-z)
Supplement: Supplementary file 1 — Supplementary Material 1 [file 12889_2026_26756_MOESM1_ESM.docx]

| Supplemental Table S1. Comparison of the sociodemographic characteristics of the Adolescent Brain Cognitive Development (ABCD) Study participants included vs. excluded in the analysis | | | |
| --- | --- | --- | --- |
| Sociodemographic characteristics | Included  (n = 4,443) | Excluded  (n = 7,519) | p |
| Age (years) (mean, 95% CI) | 11.9 (11.93, 11.98) | 12.0 (11.99, 12.02) | **<0.001** |
| Sex (%) |  |  | 0.310 |
| Female | 48.50% | 47.50% |  |
| Male | 51.50% | 52.50% |  |
| Race and ethnicity (%) |  |  | **<0.001** |
| Asian | 5.60% | 6.20% |  |
| Black | 14.50% | 23.70% |  |
| Latino/Hispanic | 14.20% | 19.00% |  |
| Native American | 3.70% | 3.30% |  |
| Other | 0.70% | 1.10% |  |
| White | 61.20% | 46.80% |  |
| Household income (%) |  |  | **<0.001** |
| Less than $75,000 | 65.60% | 59.40% |  |
| $75,000 or more | 34.40% | 40.60% |  |
| Parent's marital status (%) |  |  | **<0.001** |
| Parent married/partnered | 78.50% | 71.70% |  |
| Parent not married/unpartnered | 21.50% | 28.30% |  |
| Parent's highest education (%) |  |  | **<0.001** |
| High school education or less | 8.80% | 17.10% |  |
| College education or more | 91.20% | 82.90% |  |
| CI = confidence interval. Note: Excluded participants were missing screen time data, step count data, Year 4 cardiovascular disease risk outcomes, or Year 2 sociodemographic covariates. | | | |

| Supplemental Table S2. Associations between Year 2 screen time and step count categories and Year 4 cardiovascular disease (CVD) risk outcomes in the Adolescent Brain Cognitive Development (ABCD) Study participants, including adjustment for respective CVD outcomes at Year 2 | | | | | |  |
| --- | --- | --- | --- | --- | --- | --- |
| Screen time (hrs/day) | B (95% CI) | p | Steps/day | B (95% CI) | p |  |
| Systolic blood pressure percentile (n = 2,029) | | | | | |  |
| Low (0-4) | Reference |  | High (>12,000) | Reference |  |  |
| Medium (4-8) | -0.51 (-3.22 to 2.19) | 0.710 | Medium (6,000-12,000) | -1.51 (-5.24 to 2.22) | 0.427 |  |
| High (>8) | 0.81 (-2.32 to 3.94) | 0.612 | Low (1,000-6,000) | -0.83 (-5.40 to 3.73) | 0.720 |  |
| Diastolic blood pressure percentile (n = 2,029) | | | | | |  |
| Low (0-4) | Reference |  | High (>12,000) | Reference |  |  |
| Medium (4-8) | 2.06 (-0.61 to 4.72) | 0.130 | Medium (6,000-12,000) | **1.97 (0.44 to 4.39)** | **0.028** |  |
| High (>8) | **2.16 (-0.92 to 5.24)** | **0.036** | Low (1,000-6,000) | **6.75 (3.62 to 9.22)** | **0.003** |  |
| Hemoglobin A1c (%) (n = 238) | | | | | |  |
| Low (0-4) | Reference |  | High (>12,000) | Reference |  |  |
| Medium (4-8) | 0.01 (-0.07 to 0.08) | 0.545 | Medium (6,000-12,000) | 0.02 (-0.05 to 0.09) | 0.514 |  |
| High (>8) | -0.08 (-0.16 to 0.003) | 0.331 | Low (1,000-6,000) | 0.06 (-0.02 to 0.14) | 0.161 |  |
| Total cholesterol (mg/dL) (n = 241) | | | | | |  |
| Low (0-4) | Reference |  | High (>12,000) | Reference |  |  |
| Medium (4-8) | -0.54 (-8.04 to 6.97) | 0.888 | Medium (6,000-12,000) | -2.52 (-13.64 to 8.61) | 0.657 |  |
| High (>8) | 0.89 (-7.37 to 9.14) | 0.833 | Low (1,000-6,000) | -2.29 (-15.19 to 10.62) | 0.727 |  |
| HDL cholesterol (mg/dL) (n = 272) | | | | | |  |
| Low (0-4) | Reference |  | High (>12,000) | Reference |  |  |
| Medium (4-8) | 0.71 (-2.02 to 3.45) | 0.608 | Medium (6,000-12,000) | -4.11 (-6.21 to -2.01) | 0.050 |  |
| High (>8) | -0.23 (-3.34 to 2.88) | 0.885 | Low (1,000-6,000) | **-4.27 (-7.21 to -3.01)** | **0.036** |  |
| Non-HDL cholesterol (mg/dL) (n = 241) | | | | | |  |
| Low (0-4) | Reference |  | High (>12,000) | Reference |  |  |
| Medium (4-8) | 0.71 (-2.02 to 3.45) | 0.112 | Medium (6,000-12,000) | 3.96 (1.76 to 4.24) | 0.050 |  |
| High (>8) | **1.23 (0.96 to 2.88)** | **0.002** | Low (1,000-6,000) | **4.72 (3.43 to 5.98)** | **0.020** |  |
| All models include screen time and physical activity (step count) as the joint independent variables and were adjusted for Year 2 age, sex, race/ethnicity, household income, parental educational level, parental marital status, calendar month, data collection period (i.e., before or during the COVID-19), and respective Year 2 blood pressure, hemoglobin A1c, or cholesterol values. Participants with a prior diagnosis of diabetes were excluded from the analysis of hemoglobin A1c and participants on hypertension medications were excluded from analyses of systolic and diastolic blood pressure. **Bold** indicates statistical significance at p<0.05. | | | | | |  |
|  |  |  |  |  |  |  |
|  |  |  |  |  |  |  |

| Supplemental Table S3. Associations between self-reported total recreational screen time and step count categories and cardiovascular disease (CVD) risk outcomes in the Adolescent Brain Cognitive Development (ABCD) Study, including adjustment for body mass index (BMI) percentile | | | | | |
| --- | --- | --- | --- | --- | --- |
| Screen time (hrs/day) | B (95% CI) | p | Steps/day | B (95% CI) | p |
| Systolic blood pressure percentile | | | | | |
| Low (0-4) | Reference |  | High (>12,000) | Reference |  |
| Medium (4-8) | -1.04 (-2.97 to 0.90) | 0.294 | Medium (6,000-12,000) | 1.97 (-0.45 to 2.52) | 0.121 |
| High (>8) | 1.04 (-1.15 to 3.23) | 0.353 | Low (1,000-6,000) | 2.84 (-0.93 to 4.75) | 0.098 |
| Diastolic blood pressure percentile | | | | | |
| Low (0-4) | Reference |  | High (>12,000) | Reference |  |
| Medium (4-8) | 1.96 (-0.28 to 3.49) | 0.096 | Medium (6,000-12,000) | **1.97 (0.44 to 4.39)** | **0.011** |
| High (>8) | **3.20 (0.54 to 4.80)** | **0.014** | Low (1,000-6,000) | **5.93 (4.93 to 7.94)** | **<0.001** |
| Hemoglobin A1c (%) |  |  |  |  |  |
| Low (0-4) | Reference |  | High (>12,000) | Reference |  |
| Medium (4-8) | -0.017 (-0.07 to 0.04) | 0.545 | Medium (6,000-12,000) | 0.02 (-0.05 to 0.091) | 0.514 |
| High (>8) | -0.031 (-0.09 to 0.03) | 0.331 | Low (1,000-6,000) | 0.06 (-0.024 to 0.14) | 0.161 |
| Total cholesterol (mg/dL) |  |  |  |  |  |
| Low (0-4) | Reference |  | High (>12,000) | Reference |  |
| Medium (4-8) | -3.96 (-8.55 to 0.63) | 0.091 | Medium (6,000-12,000) | -0.90 (-2.88 to 2.43) | 0.098 |
| High (>8) | -3.74 (-9.06 to 1.58) | 0.168 | Low (1,000-6,000) | 0.94 (-0.28 to 3.17) | 0.121 |
| HDL cholesterol (mg/dL) |  |  |  |  |  |
| Low (0-4) | Reference |  | High (>12,000) | Reference |  |
| Medium (4-8) | -0.32 (-2.18 to 1.53) | 0.732 | Medium (6,000-12,000) | **-1.66 (-3.75 to -0.07)** | **0.005** |
| High (>8) | -2.31 (-3.47 to 0.84) | 0.232 | Low (1,000-6,000) | **-3.14 (-6.23 to -0.23)** | **0.035** |
| Non-HDL cholesterol (mg/dL) | | | | | |
| Low (0-4) | Reference |  | High (>12,000) | Reference |  |
| Medium (4-8) | 0.82 (-1.08 to 1.53) | 0.067 | Medium (6,000-12,000) | -0.86 (-2.75 to 0.87) | 0.089 |
| High (>8) | 2.42 (-0.47 to 3.40) | 0.145 | Low (1,000-6,000) | **4.74 (-0.23 to 6.23)** | **0.047** |
| All models include screen time and physical activity (step count) as the joint independent variables and were adjusted for Year 2 age, sex, race/ethnicity, BMI percentile, household income, parental educational level, parental marital status, calendar month, and data collection period (i.e., before or during the COVID-19). Participants with a prior diagnosis of diabetes were excluded from the analysis of hemoglobin A1c and participants on hypertension medications were excluded from analyses of systolic and diastolic blood pressure. **Bold** indicates statistical significance at p<0.05. | | | | | |

| Supplemental Table S4. Associations between self-reported total recreational screen time and step count categories and cardiovascular disease (CVD) risk outcomes in the Adolescent Brain Cognitive Development (ABCD) Study, including adjustment for waist circumference percentile | | | | | |
| --- | --- | --- | --- | --- | --- |
| Screen time (hrs/day) | B (95% CI) | p | Steps/day | B (95% CI) | p |
| Systolic blood pressure percentile | | | | | |
| Low (0-4) | Reference |  | High (>12,000) | Reference |  |
| Medium (4-8) | -0.95 (-2.80 to 0.90) | 0.312 | Medium (6,000-12,000) | -1.85 (-2.40 to 0.42) | 0.129 |
| High (>8) | 0.98 (-1.10 to 3.15) | 0.364 | Low (1,000-6,000) | -2.70 (-3.20 to 0.88) | 0.102 |
| Diastolic blood pressure percentile | | | | | |
| Low (0-4) | Reference |  | High (>12,000) | Reference |  |
| Medium (4-8) | 1.85 (-0.40 to 3.42) | 0.101 | Medium (6,000-12,000) | **2.88 (0.40 to 4.35)** | **0.012** |
| High (>8) | **3.05 (0.46 to 4.72)** | **0.016** | Low (1,000-6,000) | **5.68 (2.80 to 8.76)** | **<0.001** |
| Hemoglobin A1c (%) |  |  |  |  |  |
| Low (0-4) | Reference |  | High (>12,000) | Reference |  |
| Medium (4-8) | -0.016 (-0.068 to 0.036) | 0.561 | Medium (6,000-12,000) | 0.021 (-0.045 to 0.088) | 0.524 |
| High (>8) | -0.030 (-0.090 to 0.031) | 0.334 | Low (1,000-6,000) | 0.058 (-0.026 to 0.139) | 0.164 |
| Total cholesterol (mg/dL) |  |  |  |  |  |
| Low (0-4) | Reference |  | High (>12,000) | Reference |  |
| Medium (4-8) | -3.82 (-8.41 to 0.77) | 0.102 | Medium (6,000-12,000) | -0.85 (-6.72 to 2.48) | 0.110 |
| High (>8) | -3.60 (-8.90 to 1.71) | 0.179 | Low (1,000-6,000) | 0.92 (-0.31 to 3.09) | 0.125 |
| HDL cholesterol (mg/dL) |  |  |  |  |  |
| Low (0-4) | Reference |  | High (>12,000) | Reference |  |
| Medium (4-8) | -0.28 (-2.10 to 1.51) | 0.751 | Medium (6,000-12,000) | **-1.60 (-3.70 to -0.05)** | **0.041** |
| High (>8) | -2.25 (-3.45 to 0.78) | 0.240 | Low (1,000-6,000) | **-3.05 (-6.10 to -0.20)** | **0.038** |
| Non-HDL cholesterol (mg/dL) |  |  |  |  |  |
| Low (0-4) | Reference |  | High (>12,000) | Reference |  |
| Medium (4-8) | 0.75 (-1.00 to 1.48) | 0.071 | Medium (6,000-12,000) | -0.82 (-2.60 to 0.85) | 0.094 |
| High (>8) | 2.35 (-0.50 to 3.25) | 0.153 | Low (1,000-6,000) | **4.58 (-0.18 to 6.10)** | **0.049** |
| All models include screen time and physical activity (step count) as the joint independent variables and were adjusted for Year 2 age, sex, race/ethnicity, waist circumference percentile, household income, parental educational level, parental marital status, calendar month, data collection period (i.e., before or during the COVID-19), and calendar month. Participants with a prior diagnosis of diabetes were excluded from the analysis of hemoglobin A1c and participants on hypertension medications were excluded from analyses of systolic and diastolic blood pressure. **Bold** indicates statistical significance at p<0.05. | | | | | |

| Supplemental Table S5. Unadjusted associations between self-reported total recreational screen time and step count categories and cardiovascular disease (CVD) risk outcomes in the Adolescent Brain Cognitive Development (ABCD) Study | | | | | |  |
| --- | --- | --- | --- | --- | --- | --- |
| Screen time (hrs/day) | B (95% CI) | p | Steps/day | B (95% CI) | p |  |
| Systolic blood pressure percentile | | | | | |  |
| Low (0-4) | Reference |  | High (>12,000) | Reference |  |  |
| Medium (4-8) | 0.42 (-1.85, 2.69) | 0.715 | Medium (6,000-12,000) | 1.65 (-0.15, 3.45) | 0.072 |  |
| High (>8) | 1.68 (-0.89, 4.25) | 0.198 | Low (1,000-6,000) | **3.28 (0.95, 5.61)** | **0.006** |  |
| Diastolic blood pressure percentile | | | | | |  |
| Low (0-4) | Reference |  | High (>12,000) | Reference |  |  |
| Medium (4-8) | **3.45 (1.35, 5.55)** | **0.001** | Medium (6,000-12,000) | **3.82 (1.85, 5.79)** | **<0.001** |  |
| High (>8) | **5.82 (3.15, 8.49)** | **<0.001** | Low (1,000-6,000) | **8.45 (5.92, 10.98)** | **<0.001** |  |
| Hemoglobin A1c (%) |  |  |  |  |  |  |
| Low (0-4) | Reference |  | High (>12,000) | Reference |  |  |
| Medium (4-8) | -0.01 (-0.06, 0.04) | 0.635 | Medium (6,000-12,000) | 0.02 (-0.04, 0.07) | 0.525 |  |
| High (>8) | -0.02 (-0.08, 0.04) | 0.558 | Low (1,000-6,000) | 0.04 (-0.03, 0.11) | 0.238 |  |
| Total cholesterol (mg/dL) |  |  |  |  |  |  |
| Low (0-4) | Reference |  | High (>12,000) | Reference |  |  |
| Medium (4-8) | 0.01 (0.03, 1.12) | 0.258 | Medium (6,000-12,000) | 0.65 (0.25, 3.95) | 0.818 |  |
| High (>8) | 1.42 (0.85, 1.92) | 0.405 | Low (1,000-6,000) | 0.98 (0.82, 4.18) | 0.835 |  |
| HDL cholesterol (mg/dL) |  |  |  |  |  |  |
| Low (0-4) | Reference |  | High (>12,000) | Reference |  |  |
| Medium (4-8) | 0.28 (0.20, 1.79) | 0.788 | Medium (6,000-12,000) | **-4.15 (-4.90, 0.05)** | **0.056** |  |
| High (>8) | 1.58 (0.15, 2.39) | 0.228 | Low (1,000-6,000) | **-6.28 (-7.25, -5.31)** | **0.005** |  |
| Non-HDL cholesterol (mg/dL) |  |  |  |  |  |  |
| Low (0-4) | Reference |  | High (>12,000) | Reference |  |  |
| Medium (4-8) | 0.58 (-3.25, 4.41) | 0.765 | Medium (6,000-12,000) | 0.72 (0.25, 2.81) | 0.685 |  |
| High (>8) | 2.15 (-2.18, 6.48) | 0.328 | Low (1,000-6,000) | **8.85 (7.52, 9.82)** | **0.044** |  |
| All models include screen time and physical activity (step count) as the joint independent variables. The continuous step count is for 1,000 steps per day. Participants with a prior diagnosis of diabetes were excluded from the analysis of hemoglobin A1c and participants on hypertension medications were excluded from analyses of systolic and diastolic blood pressure. **Bold** indicates statistical significance at p<0.05. | | | | | |  |
|  |  |  |  |  |  |  |
|  |  |  |  |  |  |  |

**Supplemental Figure Captions**

**Supplemental Figure S1**

**Title:** Flow diagram of participant inclusion criteria


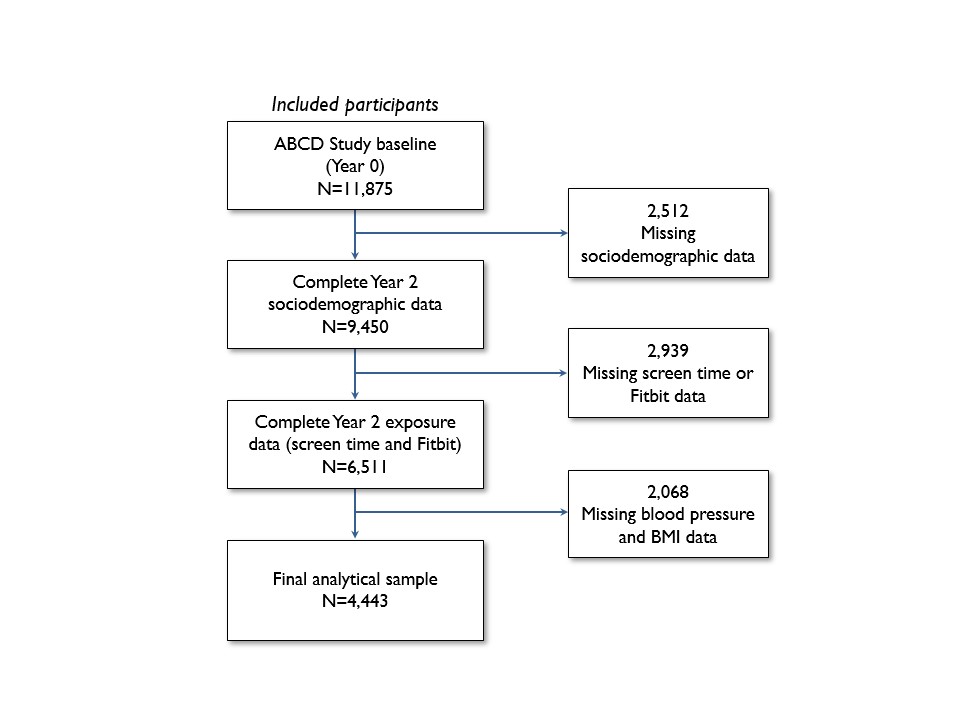


**Supplemental Figure S2.**

**Title:** Associations between self-reported screen time and step count category combinations at Year 2 and diastolic blood pressure percentile at Year 4 in 4,443 participants of the Adolescent Brain Cognitive Development (ABCD) Study, including adjustment for body mass index (BMI) percentile


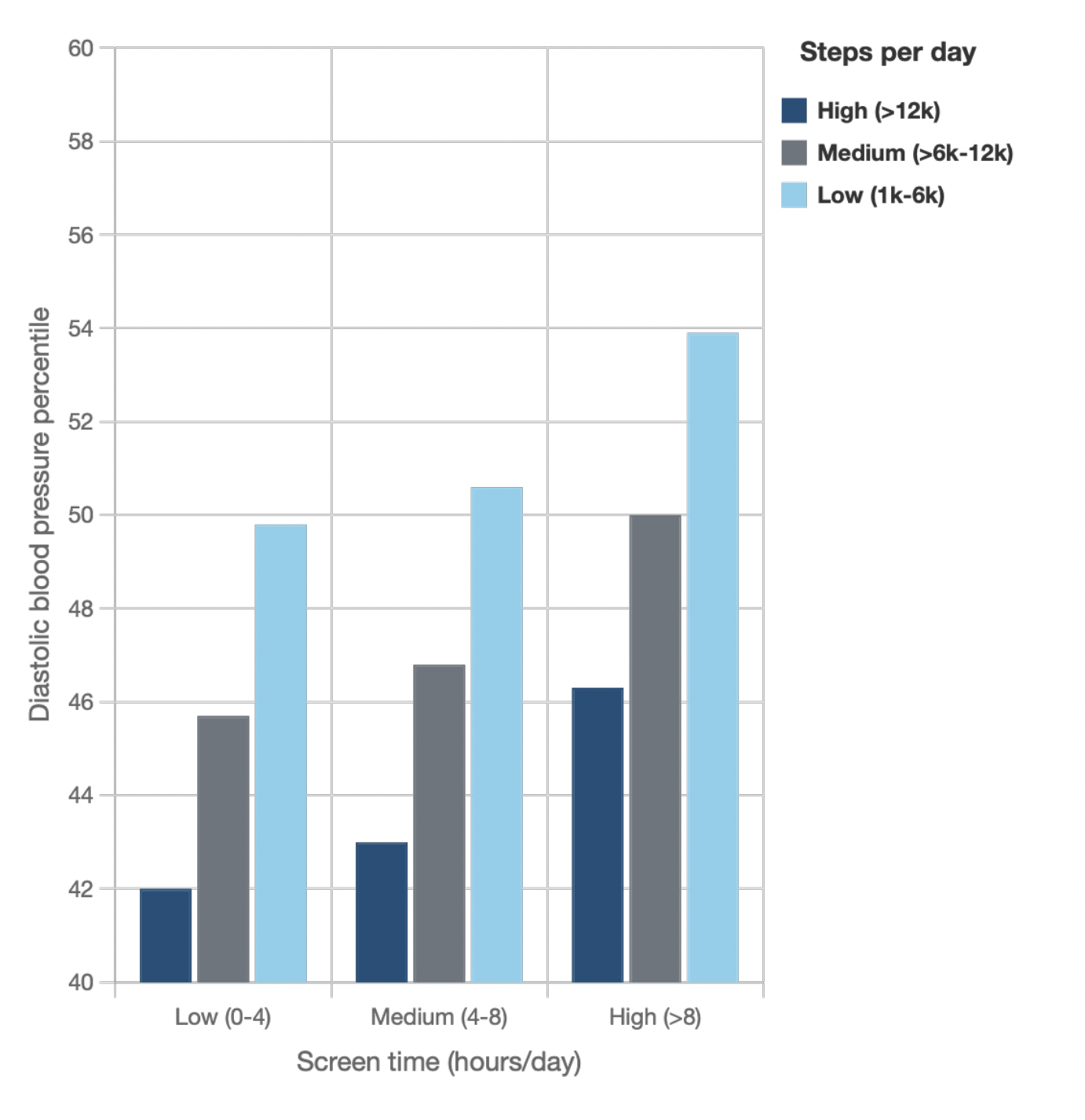


**Legend:** Results correspond to coefficients from a linear regression model with nine categories of screen time and step combinations as the independent variable and diastolic blood pressure percentile as the dependent variable, adjusting for age, sex, race/ethnicity, household income, parental education, parent marital status, BMI percentile, calendar month, and data collection period (i.e., before or during the COVID-19) at Year 2. Daily step categories included: low (1,000–6,000), medium (6,000–12,000), and high (>12,000). Daily screen time categories (hours) included: low (0–4), medium (4–8), high (>8). The low screen time and high step category was the reference category.

**Supplemental Figure S3.**

**Title:** Associations between self-reported screen time and step count category combinations at Year 2 and HDL cholesterol at Year 4 in 4,443 participants of the Adolescent Brain Cognitive Development (ABCD) Study, including adjustment for body mass index (BMI) percentile


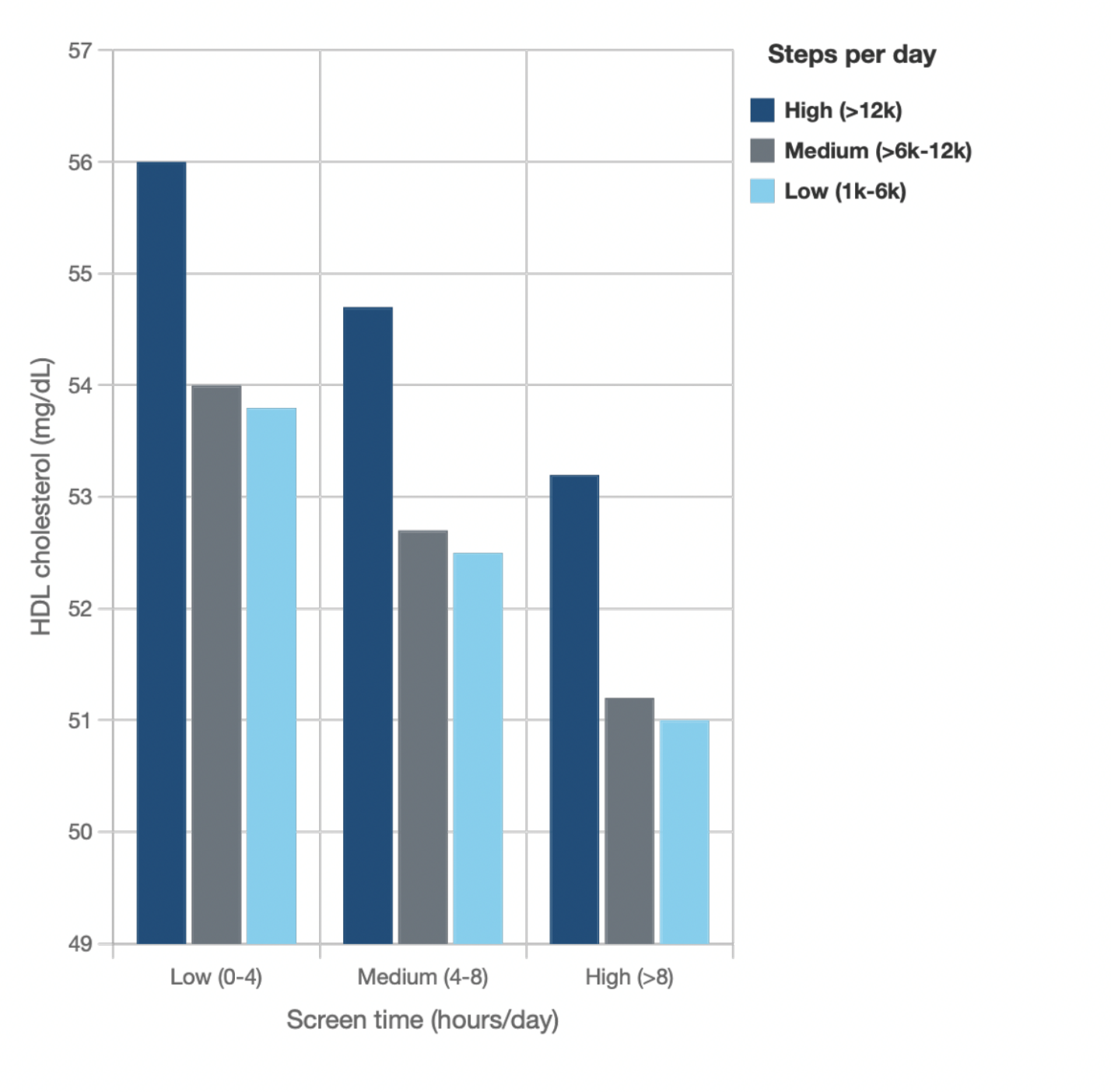


**Legend:** Results correspond to coefficients from a linear regression model with nine categories of screen time and step combinations as the independent variable and HDL cholesterol as the dependent variable, adjusting for age, sex, race/ethnicity, household income, parental education, parent marital status, BMI percentile, calendar month, and data collection period (i.e., before or during the COVID-19) at Year 2. Daily step categories included: high (>12,000), medium (6,000–12,000), and low (1,000–6,000). Daily screen time categories (hours) included: low (0–4); medium (4–8), high (>8). The low screen time and high step category was the reference category.
